# Chemoinformatics and machine learning techniques to identify novel inhibitors of the lemur tyrosine kinase-3 receptor involved in breast cancer

**DOI:** 10.3389/fmolb.2024.1366763

**Published:** 2024-04-04

**Authors:** Faris Alrumaihi

**Affiliations:** Department of Medical Laboratories, College of Applied Medical Sciences, Qassim University, Buraydah, Saudi Arabia

**Keywords:** breast cancer, LMTK3, hydrogen bonding, binding energies, WaterSwap analysis

## Abstract

Breast cancer is still the largest cause of cancer death in women, and around 70% of primary breast cancer patients are estrogen receptor (ER)-positive, which is the most frequent kind of breast cancer. The lemur tyrosine kinase-3 (LMTK3) receptor has been linked to estrogen responsiveness in breast cancer. However, the function of LMTK3 in reaction to cytotoxic chemotherapy has yet to be studied. Breast cancer therapy research remains tricky due to a paucity of structural investigations on LMTK3. We performed structural investigations on LMTK3 using molecular docking and molecular dynamics (MD) simulations of the LMTK3 receptor in complex with the top three inhibitor molecules along with a control inhibitor. Analysis revealed the top three compounds show the best binding affinities during docking simulations. Interactive analysis of hydrogen bonds inferred hotspot residues Tyr163, Asn138, Asp133, Tyr56, Glu52, Ser132, Asp313, and Asp151. Some other residues in the 5-Å region determined strong alkyl bonds and conventional hydrogen bond linkages. Furthermore, protein dynamics analysis revealed significant modifications among the top complexes and the control system. There was a transition from a loop to a-helix conformation in the protein–top1 complex, and in contrast, in complexes top2 and top3, the formation of a stabilizing sheet in the C chain was observed, which limited significant mobility and increased complex stability. Significant structural alterations were observed in the protein–top complexes, including a shorter helix region and the creation of some loop regions in comparison to the control system. Interestingly, binding free energies, including MMGB/PBSA WaterSwap analysis estimation, reveals that the top1 complex system was more stable than other systems, especially in comparison to the control inhibitor complex system. These results suggest a the plausible mode of action for the novel inhibitors. Therefore, the current investigation contributes to understanding the mechanism of action, serving as a basis for future experimental studies.

## 1 Introduction

Breast cancer is a prevalent malignant illness that primarily affects women and eventually leads to death (Albrand and Terret, 2008). Breast cancer has the second highest fatality rate after lung cancer. Breast cancer is more common in North America and Northern Europe and less common in Asia and Africa (Richie and Swanson, 2003). The female hormone estrogen is essential for the development and progression of breast cancer (Labrie et al., 1999). The proportion of estrogen receptor-a (ERa) expressed in tumor cells is two-thirds of that expressed in normal breast tissues in breast cancer (Chen et al., 2008). ERa-positive breast cancer was frequent in metastatic illness among the breast cancer types. For ERa-positive breast tumors, the estrogen signaling pathway is a primary target (Stebbing et al., 2011). Tamoxifen (antiestrogen), aromatase inhibitors (estrogen withdrawal), and fulvestrant (direct targeting on the ERa receptor) are now used to treat ERa-positive breast cancer. The main challenge in treating breast cancer is endocrine hormone resistance (Ali and Coombes, 2002).

The phosphorylation of ERa is primarily engaged in endocrine resistance via regulating transcription activity and altering stability. Endocrine resistance is more common in breast cancer patients, and it results in higher levels of Era (Rani et al., 2019). Based on SiRNA screening of multiple genes and an *in vivo* mouse model, protein kinase enzymes were discovered to be critical targets for ERa-positive cancer. Protein kinase inhibitors might be effective treatments for ERa-positive breast cancer. Serine/threonine kinases and tyrosine kinases are the two categories of protein kinase enzymes based on sequence and structural homology (Torres-Ayuso and Brognard, 2019).

Protein kinase domains are divided into 11 conserved sub-domains that fold into a tiny N-terminal lobe and a larger C-terminal lobe. The glycine-rich loop (P-loop) in the N-terminal lobe favors ATP binding, and the activation loop (T-loop) in the C-terminal lobe regulates phosphorylation activity (Robinson et al., 2000). Lemur tyrosine kinase-3 (LMTK3) is a class of serine–threonine–tyrosine kinase (Shi et al., 2014). The LMTK3 protein may have a role in the b-catenin pathway (Shi et al., 2014) and leukemic cell survival. Exon changes in human LMTK3 are responsible for its specific vulnerability to ERa-positive breast cancer (Stebbing et al., 2012). There is no exact close LMTK3 homolog in humans; however, it shows some degree of homology to the epidermal growth factor receptor (EGFR). Nevertheless, both proteins differ in detail in terms of the mechanisms by which they achieve an inactive state and prevent ATP binding. Different variants of LMTK3 are reported (VAR_057116 and VAR_028943) (Ditsiou et al., 2020). Positive selection of genes identifies LMTK3 as a novel potential biomarker for ERa-positive breast cancer (Anbarasu and Jayanthi, 2018).

In breast cancer cells, LMTK3 regulates ERa via phosphorylation activity and is directly implicated in the modulation of endocrine resistance. Furthermore, LMTK3 protects ERa from proteasomal degradation. The amount of LMTK3 is overexpressed in aggressive breast cancer and closely correlates with survival and responsiveness to hormonal therapy. Ki6713, a tumor proliferation marker, correlates with LMTK3 expression (Zhao et al., 2013). ERa is highly expressed in breast cancer, and LMTK3 phosphorylation of ERa promotes breast cancer cell proliferation, angiogenesis, migration, and progression (Xu et al., 2014).

LMTK3 inhibition in breast tumors affected ERa at two levels: mRNA production and protein stability. Targeting LMTK3 in ERa-positive breast tumors is thought to be more successful than downregulating ERa mRNA expression in cancer cells (Johnson and O’Malley, 2011). Lemur tyrosine kinase-3 (*LMTK3*; also known as *LMR3*, *TYKLM3*, and *KIAA 1883*) is a predicted dual-specificity protein kinase whose expression levels have been implicated in cancer cell invasion, endocrine resistance, poor prognosis, and overall tumor progression in different types of malignancies (Giamas et al., 2011; Zhao et al., 2013).

Computational methods have paved a new way for different approaches to finding suitable anticancer targets (Katsila et al., 2016). These approaches provide the basis for computer-aided drug design and allow having an insight into the dynamics of a suitable target (Barabási et al., 2011). Results generated from binding underwent further screening in which only a set of non-anticancer drugs were chosen. This will also enable experimentalists to directly experimentally validate the resulting compounds without requiring further validation assays. Molecular dynamics simulation was performed to verify the time-dependent behavior of the finally selected complexes and their pattern matching with the control inhibitor. This has become a widely used discipline by computational scientists and provides new insights into the time-bound behavior of biomolecules and creates measurably different simulation times, allowing to differentiate signals from different parts of a molecule (Leach, 2007) and using the MM(GB/PB)SA method to calculate binding free energies of a complex (Miller et al., 2012). This will save the cost of applying all experimental assays in repetitions and will strengthen the fact that such approaches can be applied in the future for identifying other therapeutic agents. Thus, the finding observed from the current work is a way forward in deciding the rational development of adjuvant molecules suppressing tumor formation and, in turn, addressing the curse of breast cancer.

## 2 Methodology

**FIGURE 1:**
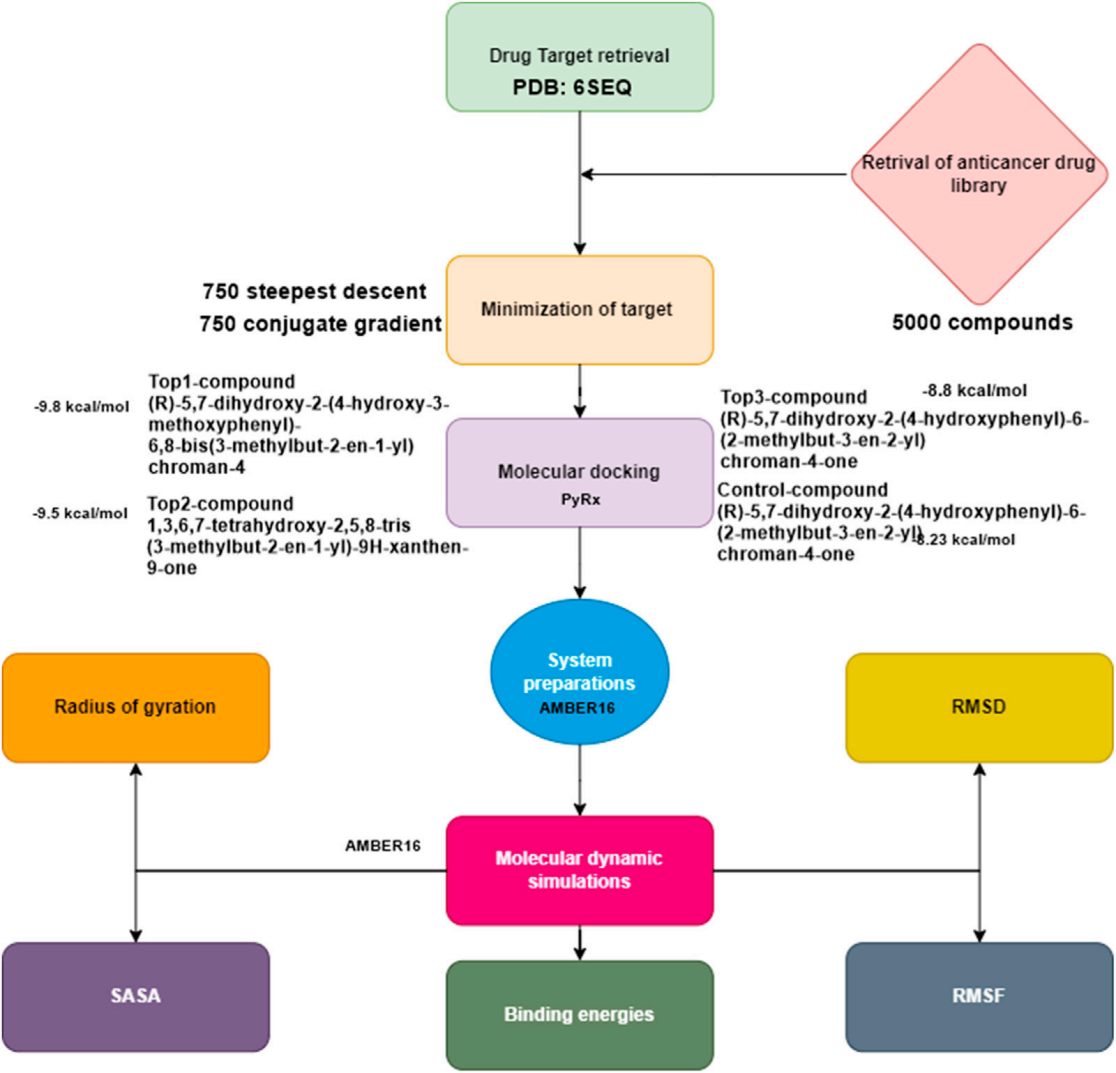
Flow chart of the current study.

The methodology depicted in Figure 1 provides a concise summary of the process for prioritizing potential drug targets. These steps enabled us to select the current target for computer-aided drug design (CADD) analysis.

### 2.1 Retrieval of ligand library

Herein, we use an anticancer library. The library was imported to PyRx 0.8 software (Dallakyan and Olson, 2015), where the library was filtered using the Lipinski rule of five. Afterward, the library was energy-minimized using an MM2 force field using ChemDraw 3D (Cousins, 2011). The energy-minimized structures were then converted into the pdbqt format to make them ready for docking.

### 2.2 Ligand preparation

The anticancer library was explored to identify potential target inhibitors. In the process of structure-based virtual screening, the library, which included inhibitors derived from natural sources, was utilized https://www.selleckchem.com/screening/fda-approved-anticancer-drug-library.html. Natural compounds, renowned for their diverse chemical scaffolds, offer a promising avenue for the discovery of novel anticancer agents with potentially reduced adverse effects. The choice to concentrate on natural inhibitors was made to harness the inherent diversity of natural products, aligning with the specific objectives of our investigation. Approximately 5,000 compounds were retrieved and were filtered against drug- and lead-likeness. Prior to that, the library was screened for drug-like inhibitors first and then lead-like inhibitors. This was accomplished using Ligand Scout 4.1’s (Wolber and Langer, 2005) drug-like and lead-like rules, which included the following drug-like rules: the five rules of Lipinski (MW ≤ 500, HBA ≤10, MLogP ≤4.15, HBD ≤5, and TPSA 40–130 Å2) (Lipinski, 2004), Veber filter (rotatable bonds ≤10, TPSA ≤140) (Veber et al., 2002), Ghose filter (LogP ≥0.4–≤ 5.6, MW ≥ 160–≤ 480, Atoms ≥20–≤ 70, and MR ≥ 40–≤ 130) (Ghose et al., 1999), Egan rule (WLogP ≤5.88 and TPSA ≤131.6) (Egan et al., 2000), and Muegge rule (TPSA ≤150, numbers of ring ≤7, numbers of carbon >4, number of heteratoms >1, HBA ≤10, MW ≥ 200–≤ 600, number of rotatable bonds ≤15, HBD ≤5, and XLogP ≥ −2–≤ 5). Filters for lead-likeness (250 MW 350, XLOGP 3.5, and rotatable bonds 7) were used to further examine drug-like compounds, as shown in Tables 1, 2. The protocol outlined in this section adheres to a widely accepted and standardized approach utilized in the screening and filtering of lead-like inhibitors against the specific protein target. This approach follows established practices in the field, ensuring consistency and comparability with other studies in the domain of lead compound discovery (Oprea, 2002).

**TABLE 1:** Physicochemical properties of the top complexes along with the control inhibitor.

Molecule	MW	Heavy atoms	Aromatic heavy atoms	Fraction Csp3	Rotatable bonds	H-bond acceptors	H-bond donors	TPSA
Top1	438.51	32	12	0.35	6	6	3	96.22
Top2	464.55	34	14	0.32	6	6	4	111.13
Top3	340.37	25	12	0.25	3	5	3	86.99
Top4	440.49	32	12	0.32	6	7	5	127.45
Top5	476.6	35	12	0.37	7	5	3	86.99
Top6	389.46	29	10	0.28	4	4	2	70.67
Top7	442.5	32	14	0.4	7	7	5	131.36
Top8	390.47	29	12	0.24	4	4	2	66.76
Control 1	356.3	26	12	0.11	3	7	0	49.85

**TABLE 2:** ADMET properties of the selected small drug-like molecules by SwissADME.

Molecule	Water	GI absorption	BBB	CYP2D6 inhibitor	Bioavailability score	Pains
	Solubility					
Molecule 1	Moderately soluble	High	No	No	0.55	0
Molecule 2	Poorly soluble	Low	No	No	0.55	1
Molecule 3	Moderately soluble	High	No	Yes	0.55	0
Molecule 4	Moderately soluble	High	No	No	0.55	1
Molecule 5	Poorly soluble	Low	No	No	0.55	0
Molecule 6	Moderately soluble	High	No	No	0.55	0
Molecule 7	Poorly soluble	Low	No	No	0.55	1
Molecule 8	Poorly soluble	High	No	No	0.55	0
Control 1	Poorly soluble	High	Yes	No	0.55	0

### 2.3 Drug target selection

Drug targets were selected based on their mechanism of function and involvement in cancer, especially in breast cancer. The best targets from the Protein Data Bank were retrieved with PDB ID: 6seq. In this study, only the available LMTK3 kinase domain was used in the structure-based virtual screening and biophysics analysis. The chosen target protein models were subjected to energy reduction to improve their quality. Herein, we did not explicitly consider post-translational modifications or delve into the broader aspects of LMTK3’s interactions with other proteins. Our study is driven by the specific research goals of identifying and characterizing potent LMTK3 inhibitors. The crystallographic experiment showed that the LMTK3 structure derived from the database exists in a monomeric form under certain circumstances. Our investigation focused on the monomeric form of LMTK3 as shown in the crystal structure, despite previous literature indicating possible dimerization under certain circumstances. Furthermore, the visualization tool UCSF Chimera (Chen et al., 2014) was used to carry out this procedure, and the structures were minimized using Gasteiger charges. Structural restrictions were removed using 1,500 rounds of minimization runs, with 750 steps using the steepest descent approach and 750 steps using the conjugate gradient method (Ahmad and Azam, 2020b). The ff03.rl force field was applied with a step size of 0.02. The minimized proteins were then submitted to a validation process to determine their quality before being used in docking investigations. The adjustments made to the cut-off values were not arbitrary or based on *ad hoc* thumb rules; rather, they are all adjusted by a comprehensive review of literature rules and further refined through optimization (Bashir et al., 2024).

### 2.4 Molecular docking

We performed virtual screening, a computer method used to identify possible lead compounds at an early stage. This technique included swiftly evaluating a large collection of anticancer drugs via computer-based algorithms and simulations. Virtual screening of the ligand library against LMTK3 was performed using PyRx (Dallakyan and Olson, 2015). To account for protein flexibility, especially in regions with disorder, we leveraged various sources of structural information. Experimental crystal structures, obtained from reliable databases https://www.rcsb.org/structure/6SEQ, played a pivotal role in providing insights into the potential conformations of the protein, encompassing both ordered and disordered regions (Stebbing et al., 2018). This approach allowed us to consider the inherent flexibility of the protein and enhance the realism of our docking simulations. The docking coordinates set depend on the active site information obtained from the active site prediction phase. The coordinates of an active site residue Asn313 x = −19.236 y = 23.590 and z = −56.880F were applied during the docking study for each compound. The number of iterations carried out is 100. Each docking conformation was assigned with a binding energy score in kcal/mol. The one with the lowest energy score was considered the best binder and complexed with the enzyme for further investigation. The visualization of the best docked complexes was carried out using UCSF Chimera v1.15 and Discovery Studio v.2020 (Biovia, 2017).

### 2.5 Molecular dynamics simulations

The starting conformation for the molecular dynamics (MD) simulations was based on the coordinates from the Protein Data Bank (PDB) and was prepared for simulation using the AMBER16 simulation program (Case et al., 2016)**.** The initial parameterization was carried out using the antechamber program of AMBER. The force field that was used is FF14SB (Case et al., 2014) for the enzyme, while for ligands, GAFF was used (Sprenger et al., 2015). The simulation was performed in the following order: energy minimization of the complexes, heating, equilibrium, and production run of at least 300 ns. Temperature control was achieved via Langevin dynamics. SHAKE algorithm was applied for constraining hydrogen bonds (Kräutler et al., 2001). Simulation trajectories were evaluated through the CPPTRAJ program (Roe et al., 2013). The coordinates of alpha carbon (C) are commonly thought to indicate an amino acid’s location in three-dimensional space. RMSD is a metric that allows comparing the relative locations of protein C atoms by computing their averaged distance over a period. It is written mathematically as

$$\text{RMSD}=\sqrt{\frac{1}{N}\sum_{i}d^{2}i},$$ (2.1)

where N is the number of compared atoms and d_i_ is the distance between the *i*th pair of atoms.

Another key metric of structural changes is the RMSF. It is used to determine the backbone atoms of the docked target (N, C, and C). It is the root mean square of the averaged distance between an atom and its average geometric location in a particular set of dynamics, and it may be read as the set of atom positions recorded over a specific time scale. The RMSF is calculated using the following equation:

$$\text{RMSF}=\sqrt{\begin{array}{c} \sum \text{Tt}\text{k}\left(\text{Xi}\left(tk\right)-X\right)\\ /T \end{array}},$$ (2.2)

where T represents the time interval, x_i_ represents the position of an atom at a particular time, and x represents the averaged position of the atom.

The radius of gyration is used to assess the overall packing quality and density of a structure. It is a physical characteristic that may be estimated experimentally, most commonly via small-angle X-ray scattering (SAXA). The following equation was used to quantify the compactness of a macromolecular system:

$$\text{Rg}=Ʃ\text{iN}=1\text{mi}\left(\text{ri}-\text{rcm}\right)2/\;Ʃ\text{iN}=1\text{ mi},$$ (2.3)

where N is the total number of atoms, mi denotes the mass of atom I, ri denotes the position vector of atom I, and rcm denotes the molecule’s center of mass.

### 2.6 Solvent-accessible surface area analysis

The amount of solvent-accessible surface area (SASA) is critical in determining the conformation and functionalities of biological macromolecules. Typically, the amino acid residues on a protein’s surface operate as active sites and/or interact with other molecules and ligands. This allows researchers to obtain a better understanding of the molecule’s behavior in a solvent environment, distinguishing between its hydrophilic and hydrophobic properties and revealing the components involved in protein–ligand interactions (Gromiha and Ahmad, 2005).

### 2.7 Binding free energy estimation

The AmberTools 16 software’s MMPBSA.py module was utilized to calculate various binding free energies for the system [31]. The main objective of this study was to assess the differences in free energy between the complex in two different phases: solvated and gas phases. The total binding free energy was determined using the following approach:

$$\Delta G\text{ binding free energy}=\Delta G\text{ bind},\text{vaccum}+\Delta G\text{ solv},\text{complex }–\;\left(\Delta G\text{ solv},\text{ligand}+\Delta G\text{ solv},\text{receptor}\right).$$ (2.4)

Molecular mechanics generalized Born surface area (MMGBSA) and molecular mechanics Poisson–Boltzmann surface area (MMPBSA) were used to choose 100 frames from simulated trajectories. To investigate the function of key amino acids in inhibitor interactions, delta free energy was deconstructed into protein residues.

### 2.8 WaterSwap analysis

WaterSwap was carried ou**t** to calculate the binding energies of the complex along with solvated systems inside the cavity. WaterSwap samples conformational space using MC simulations [using the Sire program (Woods et al., 2014)]. To begin the MC computation, a starting configuration is necessary, which is commonly obtained via MD simulations of the protein–ligand complex (Ahmad and Azam, 2020a). WaterSwap computations were used to construct the most representative structures from the ensemble obtained by the MD simulations. The free energy was then calculated for all three complex systems along with the control system using the free energy perturbation (FEP) (Zwanzig, 2004), thermodynamic integration (TI) (Oostenbrink et al., 2000), and Bennett acceptance ratio (BAR) (Bennett, 1976) methods. Three approaches were chosen to calculate free energy because the similarity of the energy values obtained from these methods indicates the dependability and convergence of the results.

## 3 Results

### 3.1 Physicochemical characteristics

The top compounds from the library were screened and further tested for ADMET characteristics. All the compounds in each library had already been subjected to Lipinski’s rule of five and ADMET descriptions prior to docking. If the medicine meets the physiochemical property requirements of Lipinski’s rule of five, it will be declared optimum. This test assesses if a chemical compound is drug-like, defined as having biological action that may be administered orally. A drug-like chemical molecule should contain hydrogen bond donors (HBDs) > 5, molecular weight (MW) 500 g/mol, and hydrogen bond acceptors (HBAs) sites >10, and log *p*-value 5 reflects hydrophobicity of a compound, according to the rule of thumb (RO5). Furthermore, two new factors were added: rotatable bonds 10 and a polar surface area (PSA) 140 A, both of which are concurrent with drug flexibility and permeability. SwissADME (Daina et al., 2017) was used to determine ADMET descriptors to compute absorption, distribution, metabolism, elimination, and toxicity (ADMET). The ADMET blood–brain barrier was used in this study. The ADMET plasma protein binding descriptor was used to determine if a medication molecule will form a strong link with the blood carrier protein. The ADMET CYP2D6 binding model was used to evaluate cytochrome P450 2D6 enzyme inhibition using a compound’s 2D chemical structure. ADMET hepatotoxicity assesses the likelihood of hepatotoxicity proliferation in humans caused by a wide range of structurally varied substances. Compounds should correspond with the needs of AlogP98 5 and PSA 140 2 for good cell permeability. Tables 1, 2 provide the Lipinski and ADMET descriptions of the top eight complexes along with the control molecule.

### 3.2 Molecular docking

**FIGURE 2:**
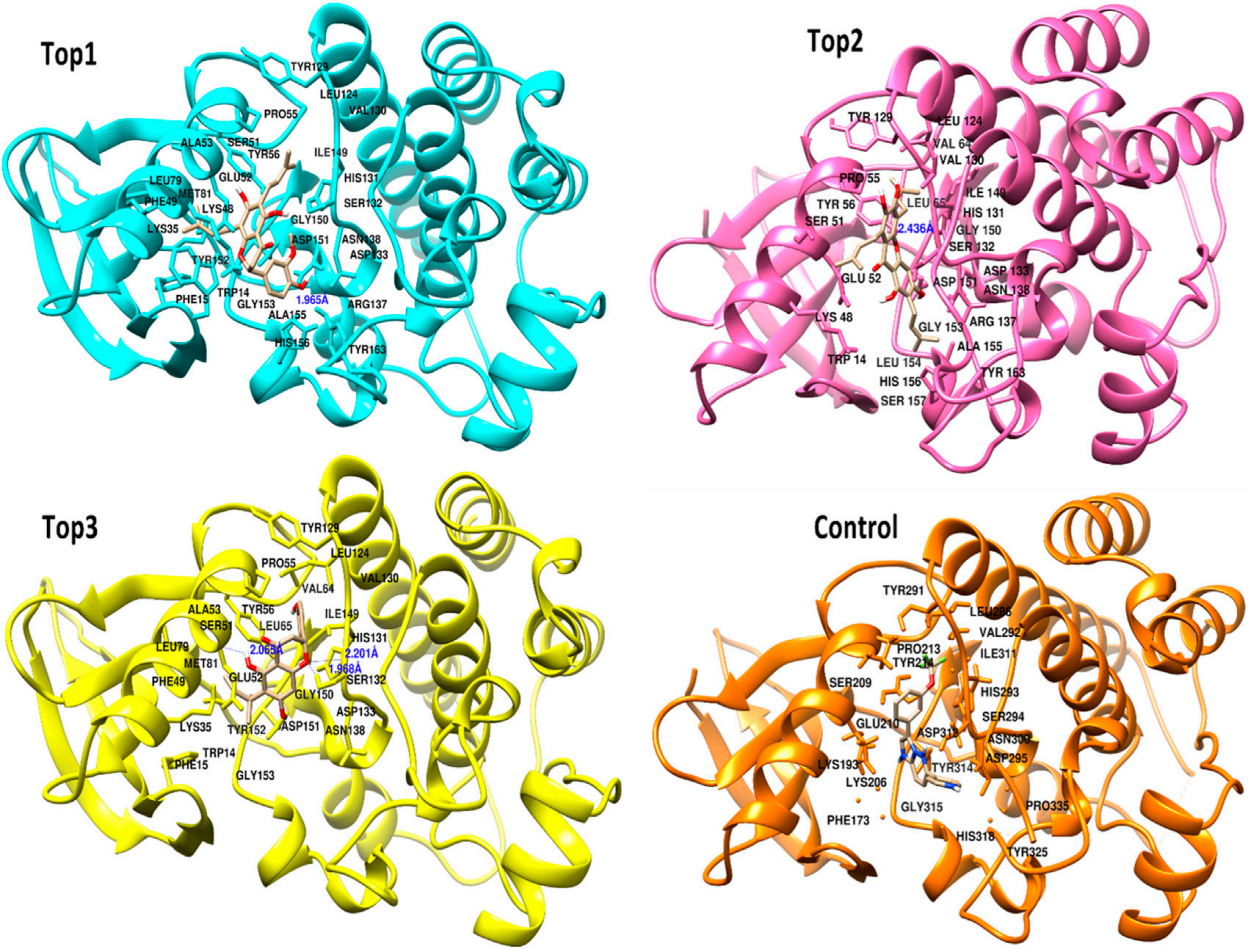
Docked poses of the top complexes along with the control inhibitor against the target protein.

**FIGURE 3:**
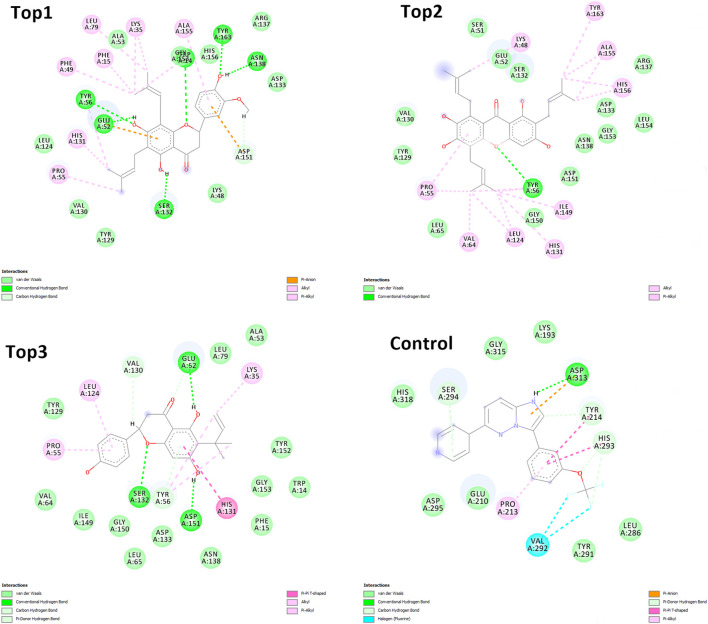
Depicting the top complexes along with the control inhibitor against the target protein.

The selected ligand molecules were docked into the active site of the target lemur tyrosine kinase-3 (LMTK3) using PyRx. Around 4,000 compounds were utilized in the docking study. The active site of the target was supported by the literature where the coordinates x = −19.236, y = 23.590, and y = −56.880 of the Asp313 were applied. After docking, the top inhibitor molecules were selected based on their binding affinities (Table 3). The top1 compound (R)-5,7-dihydroxy-2-(4-hydroxy-3-methoxyphenyl)-6,8-bis(3-methylbut-2-en-1-yl)chroman-4-one results in a high binding affinity of −9.8 kcal/mol. The top2 compound 1,3,6,7-tetrahydroxy-2,5,8-tris(3-methylbut-2-en-1-yl)-9H-xanthen-9-one has a binding affinity of −9.5 kcal/mol, and the top3 compound (R)-5,7-dihydroxy-2-(4-hydroxyphenyl)-6-(2-methylbut-3-en-2-yl)chroman-4-one has a binding affinity of −8.8 kcal/mol, whereas the control inhibitor molecule 6-(4-pyridinyl)-3-[3-(trifluoromethoxy)phenyl]imidazo [1,2-b]pyridazine results in a −8.23 kcal/mol binding affinity. Detailed visualization analysis carried out through UCSF Chimera and the preferred orientation of the ligand binding, as shown in Figure 2. The 2D interactions of the complexes were found via the Discovery Studio visualizer, which results in hydrogen bonding and other interactive linkages, as shown in Figure 3. The docked poses show the active residues of the protein-binding site. These hotspot residues, which are the active site domain residues, include Tyr163, Asn138, Asp133, Tyr56, Glu52, Ser132, Asp313, and Asp151, making strong hydrogen bonds during the docking simulation. Furthermore, validation of the hotspot residues has been investigated through molecular dynamics simulation.

**TABLE 3:** Docked score of the top complexes along with the control inhibitor against the target protein.

Compounds	IUPAC names	2D structure	Binding affinities (kcal/mol)
Top1	(R)-5,7-dihydroxy-2-(4-hydroxy-3-methoxyphenyl)-6,8-bis(3-methylbut-2-en-1-yl)chroman-4-one	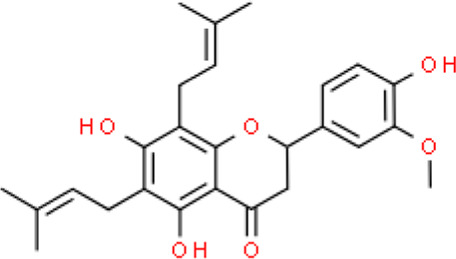	−9.8
Top2	1-Hydroxy-3,6,7-trimethoxy-2,8-bis(3-methyl-2-buten-1-yl)-9H-xanthen-9-one	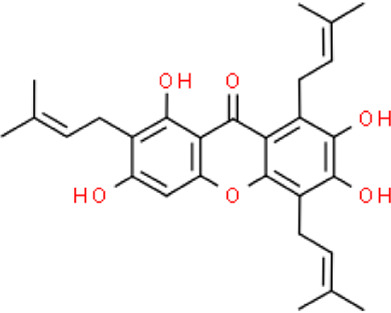	−9.5
Top3	(2S)-5,7-dihydroxy-2-(4-hydroxyphenyl)-6-(2-methyl-3-buten-2-yl)-2,3-dihydro-4H-chromen-4-one	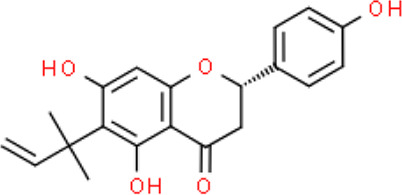	−8.8
Top4	(Z)-2,6-dimethoxy-7-(4-methylpent-3-en-1-yl)-9-(4-methylpent-3-en-1-ylidene)-9H-xanthene-3,8-diol	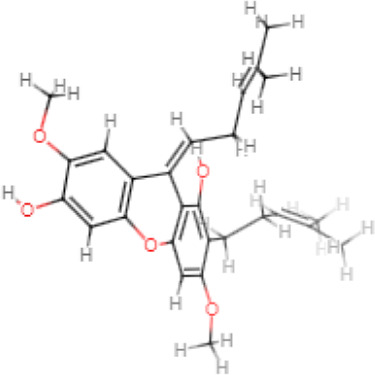	−8.7
Top5	(2S)-2-(3,4-dihydroxyphenyl)-5,7-dihydroxy-6-(2-hydroxy-3-methyl-3-buten-1-yl)-8-[(1E)-3-methyl-1-buten-1-yl]-2,3-dihydro-4H-chromen-4-one	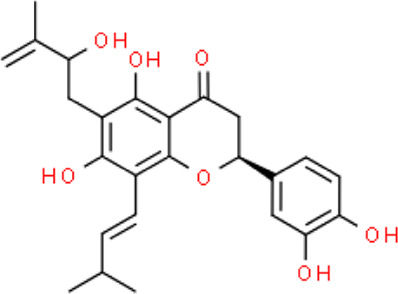	−8.32
Top6	(2S)-5,7-dihydroxy-2-[4-hydroxy-3,5-bis(3-methyl-2-buten-1-yl)phenyl]-8-(3-methyl-2-buten-1-yl)-2,3-dihydro-4H-chromen-4-one	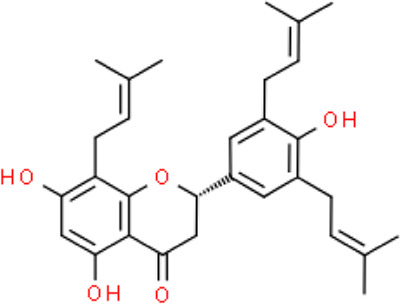	−8.30
Top7	11-Hydroxy-1-isomangostin	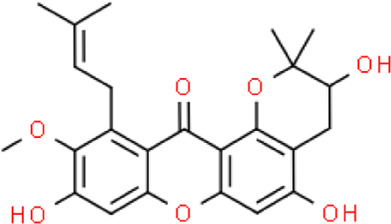	−8.24
Top8	7-Hydroxy-3-[4-hydroxy-3-(3-methyl-2-buten-1-yl)phenyl]-8-(3-methyl-2-buten-1-yl)-4H-chromen-4-one	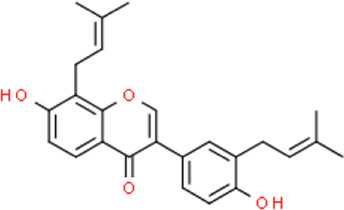	−8.23
Control	6-(4-Pyridinyl)-3-[3-(trifluoromethoxy)phenyl]imidazo [1,2-b]pyridazine	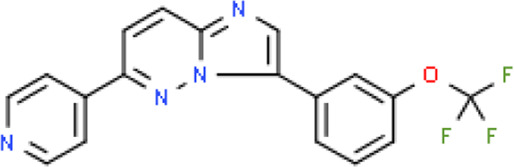	−8.23

### 3.3 Molecular dynamics simulations

**FIGURE 4:**
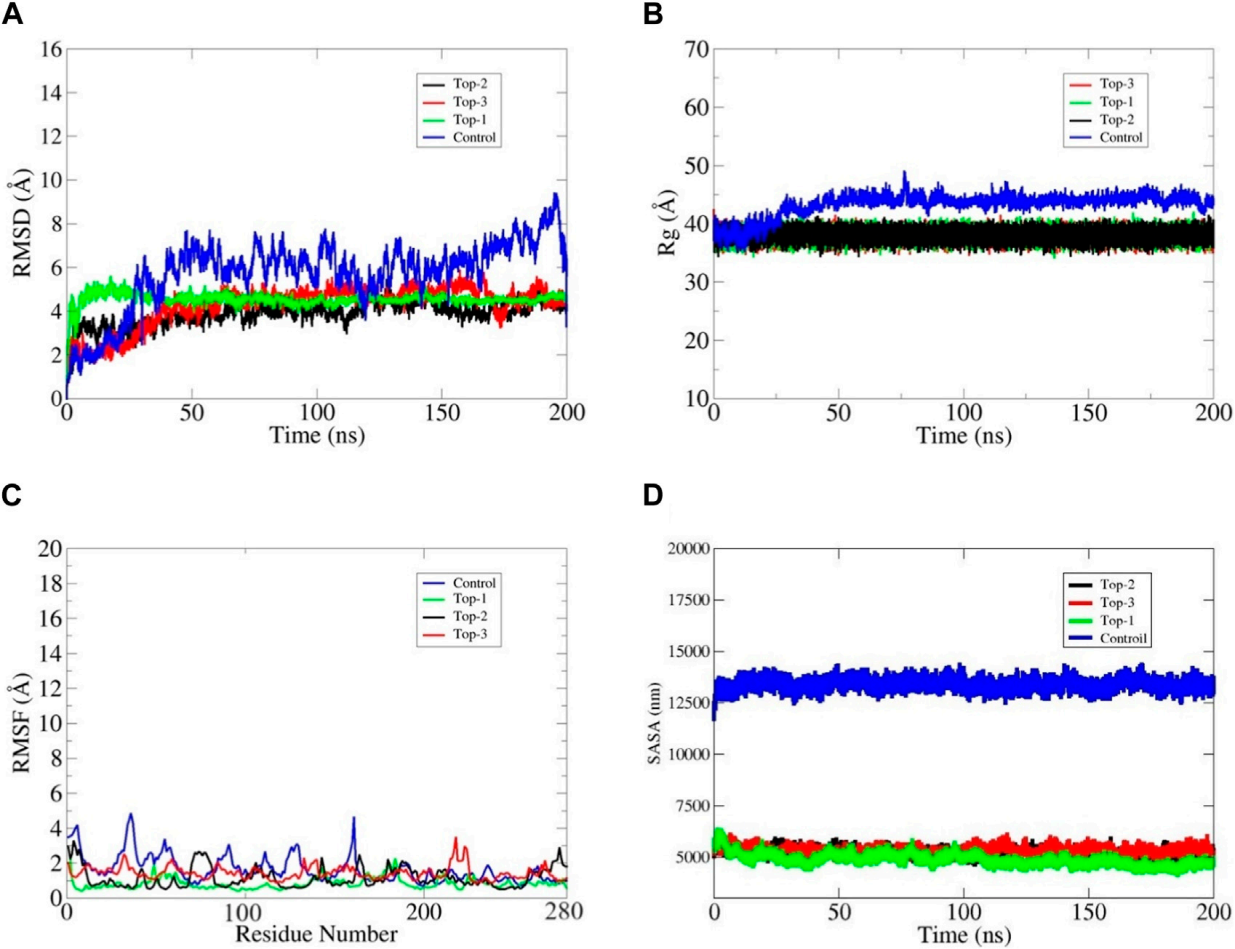
Depiction of the stability of the bound complexes, i.e., top1, top2, and top3 LMTK3 proteins in terms of **(A)** root mean square deviation (RMSD), **(B)** radius of gyration **(C)**, and root mean square fluctuation (RMSF). **(D)** SASA analysis. The structural analysis of MD simulations was performed using trajectory files, including time series values.

Structural convergence words include RMSD, RMSF, and Rg. The examination of RMSD with backbone atoms demonstrated early equilibration up to 5 ns, and after 6 ns, the structure began to converge and achieved the stable state, generating configuration with an RMSD of 4.5 MD simulation. The RMSD plot demonstrated that LMTK3 was in a stable conformation with minimal deviation from its original structure. The Cα atom changes were discovered by examining the RMSF data in connection to the LMTK3 residues. The RMSF proved that certain variations occurred in the protein’s loop regions with one exception which is flexible throughout the simulation time intervals. The root mean square deviation (RMSD) analysis was used to evaluate the docked protein’s backbone dynamics and Cα atom motions throughout a 200 ns simulation time interval. Some variations were noticed within the first 15 ns of the simulation, but the subsequent portion of the simulation indicated general stability. As illustrated in Figure 4A, the average RMSD value for the Top1 docked protein was estimated to be 4.4 Å, with a maximum peak at 5.85 Å. According to the full RMSD graph, no substantial domain alterations occurred within the physical setting of the protein–ligand combination during the top1 complex simulated system. We also investigated the top2 complex system, which remains more stable than top1 at the start of the simulation but remains a bit fluctuated during the mid-time intervals, as shown in the black line in Figure 4A. The average RMSD was recorded as 3.8 Å with a maximum value of 4.3 Å at 145 ns but remained stable till the end of the simulation. The third complex shows stability in protein at 4.5 Å, and the ligand became stable at 48 ns. The ligand shows different contacts with different residues at the start of the simulation and then it remains static in its binding cavity with some angular displacement at the end of the simulation. Rg values, which are inversely linked to protein compactness, were critical in determining ligand stability. Stable protein folding is characterized by little variation in Rg values, indicating static ligand behavior within the cavity. The rGyr values of compound top1 within the binding pocket varied from 36.8 Å to 4.0 from 0 to 200 ns and then fluctuated between 40.1 Å and 38.5 Å for all the top complexes throughout 200 ns (Figure 4B). Compound 2 had rGyr values ranging from 35.6 Å to 39Å and stabilized at 39.2 Å (Figure 4B). Compound 3, on the other hand, exhibited considerable oscillations, with rGyr values ranging from 37.7 Å to 38.6 Å throughout the simulation, indicating stable behavior (Figure 4B). On other hand, the control inhibitor shows considerable fluctuation throughout the simulation time intervals, inferring an average rGyr value of 45.6 Å with a maximum value of 49.3 Å. Considering these complexes in comparison with the control inhibitor, it has been inferred that our selected top complexes are more stable during the simulation time intervals. As shown in the figures, the control inhibitor shows more fluctuation, and the ligand inside the cavity moves inside the binding cavity with more structural changes, as seen in the RMSF graph.

The proteins’ local changes were analyzed using RMSF, which shows an average RMSF of 1.81 Å, 2.38 Å, and 2.35 Å for complex-1, complex-2, and complex-3 for 200 ns, respectively (Figure 4C), whereas high variations were found in the N-terminal and C-terminal areas, as well as in the loop regions. The interacting binding site residues at the loop region at the C-terminus fluctuate, resulting in an open binding pocket conformation. The assessment of all the complexes at different ns revealed that complex-1 deviates by 1.4 Å from the docked complex while preserving the ligand in its binding pocket. Complexes 2 and 3 have deviations of 2.1 Å and 2.2 Å, respectively; however, this deviation and opening of the binding pocket did not allow the ligand to be retained outside the surface. However, this change has been observed in the control inhibitor, which deviates more as compared to the top complexes; herein, the control shows more deviation with a maximum RMSF value of 5 Å, and the ligand moves around the surface of protein-binding site residues, as shown in Figure 4C. Furthermore, as shown in Figure 4, the ligand remained well positioned inside the binding site throughout the simulation and did not cause any protein destabilization among all the top complexes.

### 3.4 SASA analysis

A SASA analysis was performed to investigate the behavior of LMTK3’s hydrophilic and hydrophobic SASA. SASA findings of 5,000–5,500 nm indicated that accessibility was maintained with few modifications during the simulation and validated that the residues had been thoroughly exposed to the solvent (see Figure 4D). The findings revealed that in loop1, during the simulations, the area (182–202 residues) demonstrated less flexibility, with modest differences in a-helix and turn occurrence. The loop2 area (212–232 residues) showed more adaptability during all the complexes, but these structural modifications have been observed more fluctuated during the control complex system with a SASA value of more than 12,500 nm.

### 3.5 Binding free energy calculations

Binding energy analysis provides insight into individual residue contributions, which aids in the exploration of the protein–ligand complex’s composition. Binding energies for top1, top2, top3, and control complexes estimated by MM-GBSA are −33.74 kcal/mol, −44.07 kcal/mol, - 42.35 kcal/mol and - 26.95 kcal/mol, respectively, whereas the MM-PBSA approach projected binding energies of −33.47 kcal/mol for the top1 complex, −44.98 kcal/mol for the top2 complex, −43.09 kcal/mol for the top3 complex, and −27.41 for the control inhibitor complex, respectively. According to MM-GBSA, the van der Waals score observed (−33.63 kcal/mol) and electrostatic interactions (−12.14 kcal/mol) contribute the most to the binding energy in the top1 complex. High binding scores for the other top complexes were also investigated along the control inhibitor complex. Furthermore, gas phase and solvation phase interactions have contributed to the binding energy of all the complexes, according to MM-GB/PBSA (Table 4).

**TABLE 4:** Binding energy calculation for the top complexes along the control inhibitor after simulation time intervals of 200 ns.

Parameter	Control	Top1	Top2	Top3
MM-GBSA				
Energy van der Waals	−28.10	−33.63	−41.98	−43.17
Energy electrostatic	−10.48	−12.14	−13.97	−13.29
Total gas phase energy	−38.58	−45.77	−55.95	−56.46
Total solvation energy	11.63	12.03	11.85	14.11
Net energy	−26.95	−33.74	−44.07	−42.35
MM-PBSA				
Energy van der Waals	−28.10	−33.63	−41.98	−43.17
Energy electrostatic	−10.48	−12.14	−13.97	−13.29
Total gas phase energy	−38.58	−45.77	−55.95	−56.46
Total solvation energy	11.17	12.30	10.97	13.37
Net energy	−27.41	−33.47	−44.98	−43.09

### 3.6 WaterSwap analysis

**FIGURE 5:**
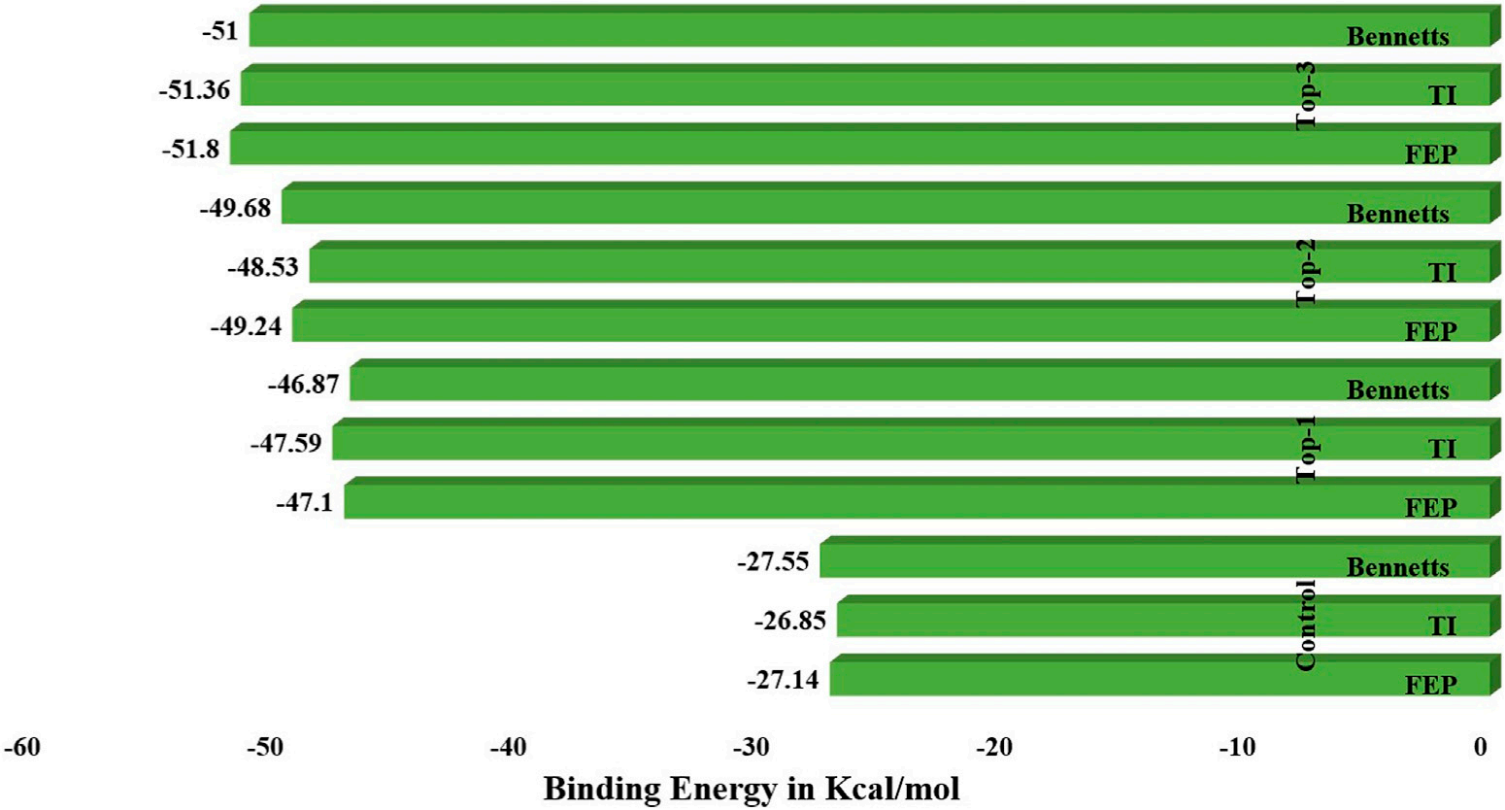
WaterSwap analysis of the simulated complex systems along with the control system.

WaterSwap analysis was used to determine the absolute binding free energy of each complex, including the control inhibitor molecule. The computed binding free energy values were shown in the WaterSwap analysis (in kcal/mol) for top inhibitors in relation to critical hotspot residues in the complex system. The computations were carried out for 1000 iterations with the cluster cut-off size set to 1 and a maximum of 25 members allowed in each cluster. A total of 1.6 109 MC motions were conducted in order to calculate the Bennett value, thermodynamic integration (TI), free energy perturbation (FEP), and TI quadrature, which are some of the approaches used. Figure 5 depicts the calculated free energy values for each complex. When the preceding procedures converge with a difference of 1 kcal/mol, the final value obtained was regarded as excellent. The drug complexes, including top1, top2, top3, and control, have an FEP value of −47.1 kcal/mol, −48.53 kcal/mol, −51.8 kcal/mol, and −27.14 kcal/mol, respectively, whereas a Ti value of −47.59 kcal/mol for top1, −48.53 kcal/mol for top2, −51.36 for top3, and −26.85 for the control system was recorded. Another approach used to find out the binding energies calculation was a BAR value for all the complexes; herein, −46.87 kcal/mol for top1, −49.68 kcal/mol for top2, −51 kcal/mol for top3, and −27.55 kcal/mol for the control system were calculated. Hence, these investigations suggest that all the top inhibitor molecules show the best binding free energies in terms of the control inhibitor system. These results further evidence and predict the scale of the potency of inhibitors for the experimental studies in a real-time system.

## 4 Discussion

Human lemur tyrosine kinase-3 (LMTK3) is a dual-specificity kinase with a well-established oncogenic involvement in several tumor types, indicating its potential as a therapeutic target. In several breast cancer cohorts, LMTK3 expression is markedly higher in high-grade breast tumors and is linked with poor survival rates (Giamas et al., 2011; Stebbing et al., 2012). Given its function in endocrine resistance, more research into its potential usefulness as a therapeutic target is necessary. In breast cancer, neoadjuvant and adjuvant chemotherapy has enhanced overall survival (Gianni et al., 2014; Hatzis et al., 2016). Resistance to cytotoxic chemotherapy, on the other hand, is the leading cause of treatment failure and mortality in women with breast cancer (Yu et al., 2013; von Minckwitz et al., 2016). An abundance of LMTK3 in human cancer was related to disease-free survival and suggested a good response to endocrine treatment. The findings also suggested that targeting LMTK3 might be useful in the treatment of drug-resistant malignancies. Kinases are phosphorylation-catalyzing enzymes that play an important part in cell activity. Kinomes are now the focus of large-scale genomics and RNA interference (RNAi) screening in drug discovery efforts, particularly in the quest for anticancer medicines (Collins and Workman, 2006).

LMTK3 deletion resulted in an 80% decrease in ERa protein in MCF-7 cells. ERa breakdown is proteasome-dependent, and inhibiting its degradation raises ERa protein levels. LMTK3 deletion decreases ERa half-life and promotes ubiquitination. LMTK overexpression, on the other hand, phosphorylates ERa, protecting it from proteasomal destruction and, therefore, stabilizing the ERa protein. LMTK3 knockdown decreased estrogen receptor 1 (ESR1) mRNA expression (Cilibrasi et al., 2021). We have used *in silico* drug design strategies to discover new inhibitors against the LMTK3 target to stop the mechanistic behavior in the cancer pathway. This is carried out via a molecular docking strategy, where we obtained top complexes with high binding affinities with strong hydrogen bond interactions. In addition, a control inhibitor was also docked against the protein LMTK3, showing strong interactions. These results were then further validated via molecular dynamics simulation studies which inferred the complexity of the top complexes, with mechanistic time interval behavior.

The comprehensive investigation of complex-1, complex-2, and complex-3 found that complex-2 is more stable because it mimics the natural secondary structural elements (SSEs). The SSE composition for each trajectory frame across the simulation duration demonstrates that all three top complexes are structurally stable.

The mean RMSD for the top1 was 4.4 Å, with a maximum value of 5.85 Å. We also looked at top2 complex systems, which are more stable than top1 at the start of the simulation but are more fluctuated over the mid-time intervals, as shown by the black line in Figure 4A. The average RMSD was 3.8, with a maximum of 4.3 at 145 ns, although it remained constant until the end of the experiment. The third compound demonstrates protein stability at 4.5 and ligand stability at 48 ns. The ligand makes distinct interactions with different residues at the start of the simulation and then remains static in its binding cavity with some angular movement at the conclusion. Rg values were crucial in assessing ligand stability since they are inversely related to protein compactness. The RMSF value also determined the thermal flexibility of the system during the simulation time intervals, which inferred that the top1 complex was more stable as compared to the control system. The other systems were also stable during simulations with a bit of fluctuation at some time intervals but remained static at the end of the simulation. Following the trajectory analysis, the top two compounds were subjected to atom contact parallel to SASA to determine non-native atom contacts as well as ligand–solvent interactions. Furthermore, a unique and more advanced WaterSwap approach was employed to cross-verify the MMGB/PBSA results. WaterSwap agrees on the inhibitor affinity for the receiver domain, as evidenced by the minimal convergence of the Bennett, TI, and FEP algorithms.

These investigations further open a way toward pilot-scale experimental analysis for further validation of compounds against cancer therapies.

## 5 Conclusion

In the current study, a potential drug candidate lemur tyrosine kinase-3 (LMTK3) revealed a small drug inhibitor molecule. The aim of this study was to identify potential therapeutic candidates by molecular docking. The molecular docking protocol was used to search for inhibitors targeting the therapeutic target. Functional residues (Tyr163, Asn138, Asp133, Tyr56, Glu52, Ser132, Asp313, and Asp151) from the active pocket of the enzyme were shown to contribute to strong hydrogen bonds with the compound. The computational predictions of hotspot residues with their behavior in dynamic simulations increase the reliability of our results. The detected hotspot residues continuously maintained their binding behavior throughout the simulation, indicating a strong and persistent interaction interface. Furthermore, molecular dynamics simulation illustrated the formation of stable complex with no observed global conformational changes. Binding free energy estimation highlighted key contributions from van der Waals and less from solvation energy. Additionally, a major change in the binding energies of the mentioned functional residues was found compared to the control inhibitor during simulation time intervals. We expect that the findings of this study are promising and may contribute to the identification of biological active leads. The results of the MD simulations exhibited all the top complexes with the highest potential as an inhibitor against LMTK3. Additionally, the top1 complex displayed strong binding affinities. Furthermore, this investigation will lead our experimentalist toward *in vivo* and *in vitro* studies, which will further enhance the drug substance toward a drug product.

## Data Availability

The original contributions presented in the study are included in the article/Supplementary Material; further inquiries can be directed to the corresponding author.

## References

[B1] Ahmad F. Azam S. S. (2020a). From pan-genome to protein dynamics: a computational hierarchical quest to identify drug target in multi-drug resistant Burkholderia cepacia. J. Mol. Liq. 317, 113904. 10.1016/j.molliq.2020.113904

[B2] Ahmad F. Azam S. S. (2020b). Role of ring positioning and preferential occupation of ligand obtained through molecular dynamics simulation of peptidoglycan associated lipoprotein (Pal). J. Mol. Graph. Model. 98, 107585. 10.1016/j.jmgm.2020.107585 32304985

[B3] Albrand G. Terret C. (2008). Early breast cancer in the elderly: assessment and management considerations. Drugs Aging 25, 35–45. 10.2165/00002512-200825010-00004 18184027

[B4] Ali S. Coombes R. C. (2002). Endocrine-responsive breast cancer and strategies for combating resistance. Nat. Rev. Cancer 2, 101–112. 10.1038/nrc721 12635173

[B5] Anbarasu K. Jayanthi S. (2018). Identification of curcumin derivatives as human LMTK3 inhibitors for breast cancer: a docking, dynamics, and MM/PBSA approach. 3 Biotech. 8, 228. 10.1007/s13205-018-1239-6 PMC5924428 29719770

[B6] Barabási A. L. Gulbahce N. Loscalzo J. (2011). Network medicine: a network-based approach to human disease. Nat. Rev. Genet. 12, 56–68. 10.1038/nrg2918 21164525 PMC3140052

[B7] Bashir Y. Noor F. Ahmad S. Tariq M. H. Qasim M. Tahir ul Qamar M. (2024). Integrated virtual screening and molecular dynamics simulation approaches revealed potential natural inhibitors for DNMT1 as therapeutic solution for triple negative breast cancer. J. Biomol. Struct. Dyn. 42, 1099–1109. 10.1080/07391102.2023.2198017 37021492

[B8] Bennett C. H. (1976). Efficient estimation of free energy differences from Monte Carlo data. J. Comput. Phys. 22, 245–268. 10.1016/0021-9991(76)90078-4

[B9] Biovia D. S. (2017). Discovery studio visualizer. San Diego, CA: USA, 936.

[B10] Case D. A. Babin V. Berryman J. T. Betz R. M. Cai Q. Cerutti D. S. (2014). The FF14SB force field. Amber 14, 29–31.

[B11] Case D. A. Betz R. M. Cerutti D. S. Cheatham T. Darden T. Duke R. E. (2016). Amber 16. San Francisco: University of California. 10.13140/rg.2.2.27958.70729

[B12] Chen G. G. Zeng Q. Tse G. M. (2008). Estrogen and its receptors in cancer. Med. Res. Rev. 28, 954–974. 10.1002/med.20131 18642351

[B13] Chen J. E. Huang C. C. Ferrin T. E. (2014). RRDistMaps: a UCSF Chimera tool for viewing and comparing protein distance maps. Bioinformatics 31, 1484–1486. 10.1093/bioinformatics/btu841 25540183 PMC4410660

[B14] Cilibrasi C. Ditsiou A. Papakyriakou A. Mavridis G. Eravci M. Stebbing J. (2021). LMTK3 inhibition affects microtubule stability. Mol. Cancer 20, 53. 10.1186/s12943-021-01345-3 33731143 PMC7968321

[B15] Collins I. Workman P. (2006). New approaches to molecular cancer therapeutics. Nat. Chem. Biol. 2, 689–700. 10.1038/nchembio840 17108987

[B16] Cousins K. R. (2011). Computer review of ChemDraw ultra 12.0. ACS Publications. 10.1021/ja204075s 21561109

[B17] Daina A. Michielin O. Zoete V. (2017). SwissADME: a free web tool to evaluate pharmacokinetics, drug-likeness and medicinal chemistry friendliness of small molecules. Sci. Rep. 7, 42717. 10.1038/srep42717 28256516 PMC5335600

[B18] Dallakyan S. Olson A. J. (2015). “Small-molecule library screening by docking with PyRx,” in Chemical biology (Springer), 243–250. 10.1007/978-1-4939-2269-7_19 25618350

[B19] Ditsiou A. Cilibrasi C. Simigdala N. Papakyriakou A. Milton-Harris L. Vella V. (2020). The structure-function relationship of oncogenic LMTK3. Sci. Adv. 6 (46), eabc3099. 10.1126/sciadv.abc3099 33188023 PMC7673765

[B20] Egan W. J. Merz K. M. Baldwin J. J. (2000). Prediction of drug absorption using multivariate statistics. J. Med. Chem. 43, 3867–3877. 10.1021/jm000292e 11052792

[B21] Ghose A. K. Viswanadhan V. N. Wendoloski J. J. (1999). A knowledge-based approach in designing combinatorial or medicinal chemistry libraries for drug discovery. 1. A qualitative and quantitative characterization of known drug databases. J. Comb. Chem. 1, 55–68. 10.1021/cc9800071 10746014

[B22] Giamas G. Filipović A. Jacob J. Messier W. Zhang H. Yang D. (2011). Kinome screening for regulators of the estrogen receptor identifies LMTK3 as a new therapeutic target in breast cancer. Nat. Med. 17, 715–719. 10.1038/nm.2351 21602804

[B23] Gianni L. Eiermann W. Semiglazov V. Lluch A. Tjulandin S. Zambetti M. (2014). Neoadjuvant and adjuvant trastuzumab in patients with HER2-positive locally advanced breast cancer (NOAH): follow-up of a randomised controlled superiority trial with a parallel HER2-negative cohort. Lancet Oncol. 15, 640–647. 10.1016/S1470-2045(14)70080-4 24657003

[B24] Gromiha M. Ahmad S. (2005). Role of solvent accessibility in structure based drug design. Curr. Comput. - Aided Drug Des. 1, 223–235. 10.2174/1573409054367664

[B25] Hatzis C. Symmans W. F. Zhang Y. Gould R. E. Moulder S. L. Hunt K. K. (2016). Relationship between complete pathologic response to neoadjuvant chemotherapy and survival in triple-negative breast cancer. Clin. Cancer Res. 22, 26–33. 10.1158/1078-0432.CCR-14-3304 26286912

[B26] Johnson A. B. O’Malley B. W. (2011). ERasing breast cancer resistance through the kinome. Nat. Med. 17, 660–661. 10.1038/nm0611-660 21647142

[B27] Katsila T. Spyroulias G. A. Patrinos G. P. Matsoukas M. T. (2016). Computational approaches in target identification and drug discovery. Comput. Struct. Biotechnol. J. 14, 177–184. 10.1016/j.csbj.2016.04.004 27293534 PMC4887558

[B28] Kräutler V. Gunsteren W. F. V. Hünenberger P. H. (2001). A fast SHAKE algorithm to solve distance constraint equations for small molecules in molecular dynamics simulations. J. Comput. Chem. 22, 501–508. 10.1002/1096-987X(20010415)22:5<501::AID-JCC1021>3.0.CO;2-V

[B29] Labrie F. Labrie C. Bélanger A. Simard J. Gauthier S. Luu-The V. (1999). EM-652 (SCH 57068), a third generation SERM acting as pure antiestrogen in the mammary gland and endometrium. J. Steroid Biochem. Mol. Biol. 69, 51–84. 10.1016/s0960-0760(99)00065-5 10418981

[B30] Leach A. (2007). Ligand-based approaches: core molecular modeling.

[B31] Lipinski C. A. (2004). Lead- and drug-like compounds: the rule-of-five revolution. Drug Discov. Today Technol. 1, 337–341. 10.1016/j.ddtec.2004.11.007 24981612

[B32] Miller B. R. McGee T. D. Swails J. M. Homeyer N. Gohlke H. Roitberg A. E. (2012). *MMPBSA.py*: an efficient program for end-state free energy calculations. J. Chem. Theory Comput. 8, 3314–3321. 10.1021/ct300418h 26605738

[B33] Oostenbrink B. C. Pitera J. W. van Lipzig M. M. Meerman J. H. van Gunsteren W. F. (2000). Simulations of the estrogen receptor ligand-binding domain: affinity of natural ligands and xenoestrogens. J. Med. Chem. 43, 4594–4605. 10.1021/jm001045d 11101351

[B34] Oprea T. I. (2002). Virtual screening in lead discovery: a viewpoint. Molecules 7, 51–62. 10.3390/70100051

[B35] Rani A. Stebbing J. Giamas G. Murphy J. (2019). Endocrine resistance in hormone receptor positive breast cancer–from mechanism to therapy. Front. Endocrinol. 10, 245. 10.3389/fendo.2019.00245 PMC6543000 31178825

[B36] Richie R. C. Swanson J. O. (2003). Breast cancer: a review of the literature. J. Insur Med. 35, 85–101. 14733031

[B37] Robinson D. R. Wu Y. M. Lin S. F. (2000). The protein tyrosine kinase family of the human genome. Oncogene 19, 5548–5557. 10.1038/sj.onc.1203957 11114734

[B38] Shi H. Li Q. Ji M. Wu J. Li Z. Zheng X. (2014). Lemur tyrosine kinase-3 is a significant prognostic marker for patients with colorectal cancer. Int. J. Clin. Exp. Pathol. 7, 1101–1107. 24695631 PMC3971314

[B39] Sprenger K. G. Jaeger V. W. Pfaendtner J. (2015). The general AMBER force field (GAFF) can accurately predict thermodynamic and transport properties of many ionic liquids. J. Phys. Chem. B 119, 5882–5895. 10.1021/acs.jpcb.5b00689 25853313

[B40] Stebbing J. Filipovic A. Ellis I. O. Green A. R. D’Silva T. R. Lenz H.-J. (2012). LMTK3 expression in breast cancer: association with tumor phenotype and clinical outcome. Breast Cancer Res. Treat. 132, 537–544. 10.1007/s10549-011-1622-z 21671015

[B41] Stebbing J. Filipović A. Giamas G. (2011). Lemur tyrosine kinase-3 (LMTK3) in cancer and evolution. Oncotarget 2, 428–429. 10.18632/oncotarget.291 21680955 PMC3248197

[B42] Stebbing J. Shah K. Lit L. C. Gagliano T. Ditsiou A. Wang T. (2018). LMTK3 confers chemo-resistance in breast cancer. Oncogene 37, 3113–3130. 10.1038/s41388-018-0197-0 29540829 PMC5992129

[B43] Torres-Ayuso P. Brognard J. (2019). Combing the cancer genome for novel kinase drivers and new therapeutic targets. Cancers (Basel) 11, 1972. 10.3390/cancers11121972 31817861 PMC6966563

[B44] Veber D. F. Johnson S. R. Cheng H. Y. Smith B. R. Ward K. W. Kopple K. D. (2002). Molecular properties that influence the oral bioavailability of drug candidates. J. Med. Chem. 45, 2615–2623. 10.1021/jm020017n 12036371

[B45] von Minckwitz G. Rezai M. Tesch H. Huober J. Gerber B. Zahm D. M. (2016). Zoledronate for patients with invasive residual disease after anthracyclines-taxane-based chemotherapy for early breast cancer – the Phase III NeoAdjuvant Trial Add-oN (NaTaN) study (GBG 36/ABCSG 29). Eur. J. Cancer 64, 12–21. 10.1016/j.ejca.2016.05.015 27323347

[B46] Wolber G. Langer T. (2005). LigandScout: 3-D pharmacophores derived from protein-bound ligands and their use as virtual screening filters. J. Chem. Inf. Model 45, 160–169. 10.1021/ci049885e 15667141

[B47] Woods C. J. Malaisree M. Michel J. Long B. McIntosh-Smith S. Mulholland A. J. (2014). Rapid decomposition and visualisation of protein–ligand binding free energies by residue and by water. Faraday Discuss. 169, 477–499. 10.1039/c3fd00125c 25340314

[B48] Xu Y. Zhang H. Lit L. C. Grothey A. Athanasiadou M. Kiritsi M. (2014). The kinase LMTK3 promotes invasion in breast cancer through GRB2-mediated induction of integrin β₁. Sci. Signal. 7, ra58. 10.1126/scisignal.2005170 24939894

[B49] Yu K.-D. Zhu R. Zhan M. Rodriguez A. A. Yang W. Wong S. (2013). Identification of prognosis-relevant subgroups in patients with chemoresistant triple-negative breast cancer. Clin. Cancer Res. 19, 2723–2733. 10.1158/1078-0432.CCR-12-2986 23549873 PMC3655097

[B50] Zhao G. Guo J. Li D. Jia C. Yin W. Sun R. (2013). MicroRNA-34a suppresses cell proliferation by targeting LMTK3 in human breast cancer MCF-7 cell line. DNA Cell Biol. 32, 699–707. 10.1089/dna.2013.2130 24050776 PMC3864372

[B51] Zwanzig R. W. (2004). High‐temperature equation of state by a perturbation method. I. Nonpolar gases. J. Chem. Phys. 22, 1420–1426. 10.1063/1.1740409

